# CuSO_4_/[Cu(NH_3_)_4_]SO_4_-Composite Thermochemical Energy Storage Materials

**DOI:** 10.3390/nano10122485

**Published:** 2020-12-11

**Authors:** Danny Müller, Christian Knoll, Georg Gravogl, Daniel Lager, Jan M. Welch, Elisabeth Eitenberger, Gernot Friedbacher, Andreas Werner, Werner Artner, Michael Harasek, Ronald Miletich, Peter Weinberger

**Affiliations:** 1 Institute of Applied Synthetic Chemistry, TU Wien, Getreidemarkt 9, 1060 Vienna, Austria; christian.knoll@gmx.at (C.K.); georg.gravogl@tuwien.ac.at (G.G.); 2 Institute of Chemical, Environmental & Biological Engineering, TU Wien, Getreidemarkt 9, 1060 Vienna, Austria; michael.harasek@tuwien.ac.at; 3 Institut für Mineralogie und Kristallographie, University of Vienna, Althanstraße 14, 1090 Vienna, Austria; ronald.miletich-pawliczek@univie.ac.at; 4 Austrian Institute of Technology GmbH, Giefinggasse 2, 1210 Vienna, Austria; daniel.lager@ait.ac.at; 5 Center for Labelling and Isotope Production, TRIGA Center Atominstitut, TU Wien, Stadionallee 2, 1020 Vienna, Austria; jan.welch@tuwien.ac.at; 6 Institute of Chemical Technologies and Analytics, TU Wien, Getreidemarkt 9, 1060 Vienna, Austria; elisabeth.eitenberger@tuwien.ac.at (E.E.); gernot.friedbacher@tuwien.ac.at (G.F.); 7 Institute for Energy Systems and Thermodynamics, TU Wien, Getreidemarkt 9, 1060 Vienna, Austria; andreas.werner@tuwien.ac.at; 8 X-ray Center, TU Wien, Getreidemarkt 9, 1060 Vienna, Austria; werner.artner@tuwien.ac.at

**Keywords:** CuSO_4_/[Cu(NH_3_)_4_]SO_4_, composite material, thermal conductivity, thermochemical energy storage, thermochemistry

## Abstract

The thermochemical energy-storage material couple CuSO_4_/[Cu(NH_3_)_4_]SO_4_ combines full reversibility, application in a medium temperature interval (<350 °C), and fast liberation of stored heat. During reaction with ammonia, a large change in the sulfate solid-state structure occurs, resulting in a 2.6-fold expansion of the bulk material due to NH_3_ uptake. In order to limit this volume work, as well as enhance the thermal conductivity of the solid material, several composites of anhydrous CuSO_4_ with inorganic inert support materials were prepared and characterized with regard to their energy storage density, reversibility of the storage reaction, thermal conductivity, and particle morphology. The best thermochemical energy storage properties were obtained for a 10:1 CuSO_4_-sepiolite composite, combining an attractive energy storage density with slightly improved thermal conductivity and decreased bulk volume work compared to the pure salt.

## 1. Introduction

The steadily increasing worldwide energy demand results in efforts to enhance energy efficiency and reduce greenhouse gas emissions [1]. In a common endeavor, most countries agreed to a renewal of joint climate targets [2,3], also associated with a significant ongoing reorientation of the energy market. Especially in industries such as steel, brick, glass, oil refining and thermal power generation, the efficient and economic use of primary energy has become a key aspect. Within this context, a report of the International Energy Agency (IEA) from 2011, stating already that an electricity production 2/3 of the invested primary energy is wasted through thermal losses [4], should be concerning to policymakers and scientists in equal measure. 

Strategies for the recovery and recycling of even a minor percentage of those enormous amounts of wasted energy could significantly contribute to enhanced energy efficiency. The crucial aim and challenge to scientific contributors herein is to provide efficient processes, capable of storing large amounts of heat [5,6,7]. In addition to heat recovery, harmonization between heat consumption and heat production would also be necessary. If, through heat storage technologies, a decoupling of production and consumption is enabled, that would constitute an important step towards a reduction in primary energy sources [8].

Various concepts for heat storage are known, some of them already operational on a technological scale, others so far only in an experimental environment. In general, three concepts of heat storage are differentiated based on the underlying principle of operation:

(1) Sensible heat storage [9,10,11]: Heat is stored by increasing the temperature of a liquid (oil, water, molten salts, …) or solid (brick, concrete, …) storage medium. To extract the stored heat, the process is reverted. Sensible heat storage is by far the simplest technologically implementable form of heat storage; thus, it is already operational on large scales in established processes. The two main drawbacks associated with this concept are the necessary heavy thermal insulation, preventing heat losses during storage through radiation, as well as the large volumes of storage medium.
(2) Latent heat storage [12,13,14]: Heat is stored taking advantage of the energy demand/energy release, associated with a phase-transfer from solid to liquid, or vice-versa. Although the first commercial applications with latent heat storage are already available, the technological readiness is less than for sensible technologies. Associated drawbacks are the necessary thermal insulation, but also the potential ageing/degradation effects of the phase change material.
(3) Thermochemical heat storage [15,16,17]. Thermochemical heat storage includes both heat stored via a reversible chemical reaction, as well as via sorption storage. Whereas sorption storage on, e.g., zeolites is a pure physical process, storage through a chemical reaction involves the reversible thermal decomposition of a (solid) storage material (*A*), liberating a reaction gas (*B*). The charged (solid) storage material (*C*) is stockpiled until, by reaction with the previously separated reaction gas, the initial discharged form of the storage material is reformed
  A_(solid)_ + ΔH↔B_(solid)_ + C_(gas)_
  Appealing advantages of this concept can be summarized as flexible operational temperature ranges, as for each application in principle a suitable storage reaction can be found; highest storage densities [18]; fast charging/discharging rates; and infinite storage, as in the absence of the reaction gas (C) no energy loss during storage can occur. Drawbacks are the notably higher technological efforts in operation, as well as the necessary development work for a custom-tailored process [5].

From a scientific point of view, the potential gains of a powerful thermochemical energy storage material outweigh its drawbacks, especially if focused on specialized applications and requirements. Such applications could request high charging/discharging frequencies, space constraints for the storage material volume and storage temperatures, as of a maximum of 350 °C. The ideal thermochemical energy storage material for this purpose would feature high storage densities, fast reaction rates and an excellent cycle stability. 

Having investigated systematically several classes of suitable thermochemical energy storage materials [19], we found transition metal ammoniates to be ideal candidates with respect to these requirements. In the literature, the coordination chemistry of ammonia for energy storage purposes has been scarcely investigated [20,21,22,23]. During a systematic study on transition metal ammoniates capable for the purpose of thermochemical storage, we investigated the reaction between CuSO_4_ and NH_3_, reversibly forming the [Cu(NH_3_)_4_]SO_4_ complex: [24] CuSO_4_ reacts in a highly exothermic manner with four molar equivalents of NH_3_, liberating a heat of reaction of 1.77 MJ kg^−1^, thus accounting to an energy storage density of 6.38 GJ m^−3^. The charging reaction is the decomposition of [Cu(NH_3_)_4_]SO_4_, restoring the initial anhydrous CuSO_4_ solid. This highly cyclically repeatable stable reaction already occurs readily and with appealing reaction rates at room-temperature. Based on these facts, several applications would be possible for ammoniates, and specifically for [Cu(NH_3_)_4_]SO_4_/CuSO_4_—e.g., in combination with solar thermal plants or thermal power plants, or as fast buffer-storage in an industrial environment. However, using the pure sulfate salt as a host storage material raises two issues which shall be addressed herein: Firstly, during the reaction with NH_3_, a significant expansion of the material occurs. This could be problematic for real applications, as this would demand extra spare volumes, or alternatively cause significant mechanical stress on the containment. Secondly, the moderate thermal conductivity of pure inorganic salts could be optimized by enhancing the heat transfer from and to the bulk storage material.

Studies on composite materials have been reported for several thermochemical energy storage materials with a focus on immobilizing or incorporating the storage material on/in a support matrix [25,26,27]. The main reason for preparing such composite materials was a confinement of the storage material during the reaction, as well as an improvement of the thermal properties. A similar concept was investigated in the present contribution for the thermochemical storage couple [Cu(NH_3_)_4_]SO_4_/CuSO_4_.

## 2. Materials and Methods 

### 2.1. Materials

CuSO_4_∙5H_2_O and the support materials were obtained commercially from Sigma-Aldrich Germany GmbH (Steinheim, Germany) (*copper(II) sulphate pentahydrate*, CAS: 7758-99-8; *silica gel 60*, CAS: 112926-00-8; *celite^®^545*, CAS: 68855-54-9; *sepiolite powder*, CAS: 63800-37-3; *vermiculite*, CAS: 1318-00-9; *molecular sieve zeolite 13X*, CAS: 63231-61-6; and *charcoal*, CAS: 7440-44-0) and used as supplied. Embottled anhydrous ammonia gas (CAS: 7664-41-7, 99.98% NH_3_) was obtained from Messer Austria GmbH (Gumpoldskirchen, Austria). 

### 2.2. Preparation of CuSO_4_ on Support-Materials

The copper(II) sulphate-loaded support materials were prepared by soaking the untreated support materials for 60 min in a saturated aqueous solution of CuSO_4_∙5H_2_O at room temperature. The soaked support materials were separated, rinsed with water and dried for 2 h at 150 °C under vacuum, before the soaking procedure was repeated. After rinsing with water, the loaded supports were dried for 2 h at 400 °C and stored after cooling in a desiccator. The amount of copper(II) sulphate loaded on the individual host carrier materials was derived gravimetrically (for sepiolite, celite and silica gel carriers) and by means of X-ray fluorescence spectroscopy (for charcoal, vermiculite, zeolite 13X carriers). Analytically determined CuSO_4_ contents are: 23 ± 2.5 wt. % on charcoal and 22 ± 4.1 wt.% on vermiculite. For zeolite 13 X a Cu^2+^-content of 6.4 ± 1.8 wt.% was determined.

For the preparation of CuSO_4_ on sepiolite, celite and silica gel 10:1 (wt. %) the necessary amount of CuSO_4_ was dissolved in H_2_O, the solid carrier material suspended in the solution at room temperature, and after stirring for 30 min, the water was evaporated. The residual solid was dried for 2 h at 400 °C and stored after cooling in a desiccator. An image of all composites before ammoniation and after the formation of the [Cu(NH_3_)_4_]SO_4_ complex is given in the supporting information, Figure S1.

### 2.3. Thermal Analysis

For thermal analysis, a Netzsch TGA/DSC 449 C Jupiter^®^ instrument (Erich Netzsch GmbH & Co. Holding KG, Selb, Germany) equipped with a water vapor furnace including an air-cooled double jacket was used. The oven operates between 25–1250 °C, regulated by an S-type thermocouple. For the determination of the energy contents at room-temperature, the NH_3_ gas flow was set to 100 mL min^−1^ allowing for a rapid and complete exchange of the atmosphere in the oven. The flow was controlled by Vögtlin Instruments “red-y” mass flow controllers (Vögtlin Instruments GmbH, Muttenz, Switzerland). The transition metal salt (10 mg) was placed in an open aluminum crucible and reacted after 10 min of stabilization under N_2_-atmosphere with NH_3_ until the exothermic reaction had ceased. The DSC was calibrated according to the procedure suggested by Netzsch, using the In, Sn, Bi, Zn, Al and Ag standards provided by the manufacturer. Before each experiment, a baseline-correction using the measurement software was performed.

For decomposition and cycling experiments, a sample mass of approximately 5 mg placed in an open alumina crucible was used, applying heating and cooling rates of 10 °C min^−1^ under a NH_3_ atmosphere created with a constant 20 mL min^−1^ flow of NH_3_ gas, controlled by Vögtlin Instruments “red-y” mass flow controllers. Prior to all measurements, a thermal correction was performed. In order to determine the decomposition (deamination) temperature of the various transition metal complexes, the fully ammoniated transition metal salt was heated to 450 °C. For the cycling experiments, after 10 min of stabilization under the NH_3_ atmosphere at room temperature, the samples were heated slightly over the earlier determined decomposition temperature, thus ensuring the complete thermal deamination of the material. Then, a cooling phase was followed under the NH_3_ atmosphere for an isothermal stabilization time of 30 min at room temperature before the heating/cooling cycle was repeated.

The evaluation of the TGA and DSC curves was performed with the Netzsch Proteus—Thermal Analysis—version 6.0.0 software package (NETZSCH-Gerätebau GmbH, Selb, Germany). Mass-losses, residual mass, DSC-values and onset temperatures were calculated from the respective area of the baseline-corrected measurement data by the software algorithms. The mass-loss is given in % and was obtained by subtracting the residual mass (in %) determined by the software analysis-algorithm from the original mass (100%). Experimental error is given according to the specifications of the instrument.

### 2.4. Scanning Electron Microscopy (SEM)

SEM images were recorded on a Quanta SEM instrument from FEI (FEI Company, Hillsboro, OR, USA) under low-vacuum and in the presence of water vapor to prevent electrostatic charge. The samples were mounted on carbon pellets on top of the sample holder and plasma vacuum deposition was used to coat the samples with a thin layer of gold to ensure appropriate conductivity. The gold coating was performed using a AGAR sputtering system at 10 mA for 30 s.

### 2.5. Nitrogen Physisorption Surface Area 

The specific surface of the samples was determined by nitrogen sorption measurements, which were performed on an ASAP 2020 (Micromeritics) instrument (Micromeritics Instrument Corporation, Norcross, GA, USA). The samples (amounts between 100–200 mg) were degassed under vacuum at 120 °C overnight (zeolite 13X was degassed at 300 °C overnight) prior to measurement. All experiments were repeated to ensure reproducibility. The surface area was calculated according to Brunauer, Emmett and Teller (BET) [28].

### 2.6. X-ray Powder Diffraction (P-XRD)

The powder X-ray diffraction measurements were carried out on a PANalytical X’Pert Pro diffractometer (Malvern Panalytical Ltd., Almelo, The Netherlands) in Bragg–Brentano geometry using Cu K_α1,2_ radiation and an X’Celerator linear detector. The recorded diffractograms were evaluated using the PANalytical program suite HighScorePlus version 4.6a (Malvern Panalytical Ltd., Almelo, The Netherlands) [29]. The XRD profiles were corrected for background and K_α2_ stripping prior to profile refinements. Phase assignment is based on the ICDD-PDF4+ database [30], the exact phase composition, shown in the conversion plots, was obtained via Rietveld-refinement incorporated in the program suite HighScorePlus v4.6a [29].

### 2.7. Transient Hot Bridge (THB)

The THB method is a transient method to measure the effective thermal conductivity λ_eff_ based on a thermoelectric sensor. The sensor technology was developed by the Physikalisch-Technische Bundesanstalt in Germany [31]. For the THB measurements, a LINSEIS THB 100 dynamic measurement system (Linseis Messgeräte GmbH, Selb, Germany) was used in combination with a kapton foil sensor with a sensor size of 42 mm × 22 mm. This sensor can be applied for a thermal conductivity range from λ = 0.01 to 1 W m^−1^ K^−1^ and a temperature range from T = −100 to 200 °C. The samples with the attached foil sensor are placed inside a lab furnace MEMMERT UFP 500, which can reach a maximum temperature of T_max_ = 250 °C under ambient atmospheric conditions. For calibration of the measured temperature T and the thermal conductivity λ, water is used as reference standard with λ(20 °C) = 0.5984 W m^−1^ K^−1^. In order to provide sufficient contact between the surface of the foil sensor and the sample itself, it is necessary to use a liquid reference due to the thickness of the metal frame around the kapton foil sensor. The sample was filled into a measuring glass cup with a volume of V ~ 100 cm^3^ and shaken under defined conditions until no optical volume change was noticed anymore. After that, the volume was measured by the measuring cup scale and weight with a lab balance. Finally, the sensor was immersed into the sample and placed inside the lab furnace.

## 3. Discussion and Results

**Figure 1:**
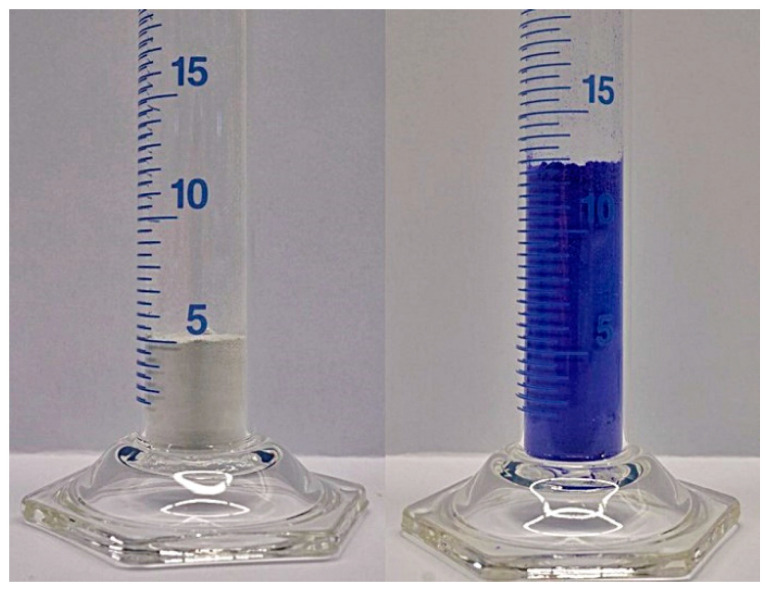
2.6-fold expansion of the bulk material during reaction of CuSO_4_ (left, white solid) with four equivalents of NH_3_, forming the [Cu(NH_3_)_4_)]SO_4_ complex (right, blue solid).

Within the scope of the systematic investigations on transition metal salts reacting with ammonia for the purpose of application to thermochemical energy storage, the reaction couple CuSO_4_ + 4 NH_3_ ↔ [Cu(NH_3_)_4_)]SO_4_ appears to be the most promising candidate for detailed experimental studies in terms of cycle stability, cycle reversibility, and energy storage density [24]. The uptake of four molar equivalents of NH_3_ is in line with a structural change on the molecular level within the structure of the crystalline solid, being responsible for a notable expansion of the material. CuSO_4_ crystallizes in the orthorhombic space group P*nma* (no. 62) with a = 8.39 Å, b = 6.69 Å, c = 4.83 Å, and a unit-cell volume of 271.10 Å^3^ (T = 273 K) for Z = 4 [32]. After the reaction with NH_3_, the resulting [Cu(NH_3_)_4_)]SO_4_ had transformed to a pseudo-orthorhombic structure, crystallizing in the monoclinic space group *P*2_1_/*c* (no. 14) with a = 14.253(3) Å, b = 7.359(2) Å, c = 14.589(3) Å, β = 91.330(6)° and a unit-cell volume of 1529.8 Å^3^ (T = 300 K) for Z = 8, as determined within this study. This molecular change is translated to a ~2.6-fold expansion of the molar volume, as exemplified by the volume expansion of the material (Figure 1).

Different studies on other thermochemical storage materials (e.g., Mg(OH)_2_ or CaCl_2_) have revealed that combination with inorganic host carrier materials forming composites could significantly enhance reaction rates. Moreover, this confines the reactive salt to the less expansive host carrier and improves the thermal conductivity within the bulk composite. In order to investigate whether the combination with a support material can improve the overall material properties of the reactive [Cu(NH_3_)_4_)]SO_4_/CuSO_4_ couple, a deposition on various carrier materials has been considered for this study—i.e., charcoal, silica gel, celite, zeolite 13X, sepiolite, and vermiculite. Charcoal was considered for its enhanced adsorption capacity, due to the internal spaces and pores within the lightweight carbon residue material. The silica gel chosen for this study represents a nanoporous non-crystalline SiO_2_ with average pore sizes around 60 ± 10 Å. Celite is calcinated amorphous opal-based silicious material, made of highly microporous diatomaceous earth originating from microalgae skeleton fabrics. Zeolite X13 is in contrast a crystalline molecular sieve, a faujasite-type alkali–alumosilicate framework compound, i.e., Na_43_(Al_43_Si_53_O_192_).nH_2_O, structurally consisting of 10 Å sodalite-type cages within the cubic framework arrangement [33]. Vermiculite is, in contrast, related to hydrous trioctahedral mica, a hydrated (Mg,Fe)_3_(OH)_2_[(Al,Si)_4_O_10_]·4H_2_O phyllosilicate with double sheets of H_2_O interleaved with silicate layer units [34]. Sepiolite is a fibrous phyllosilicate of the composition Mg_8_(OH)_4_[Si_12_O_30_]·12H_2_O with inverted tetrahedral sheets that form 4 × 11 Å channel units along the fibre axis. 

Charcoal, vermiculite and zeolite 13X were selected according to their micro- and nanoporous properties and sub-nanoscaled structural features with respect to the capability of CuSO_4_ intercalation. CuSO_4_ being diluted within the various forms of more-or-less porous silica and sepiolite carriers was studied, to derive whether the inert carrier materials are capable of compensating for the bulk expansion to a certain degree, whilst retaining the appealing energy density of the pure salt. The selected host compounds and carrier materials were purchased in the highest obtainable purities and characterized by powder X-ray diffraction and by nitrogen physisorption. The results for the measured specific surfaces of the materials are given in Table 1.

### 3.1. Reaction Enthalpy and Cycle Stability

**Figure 2:**
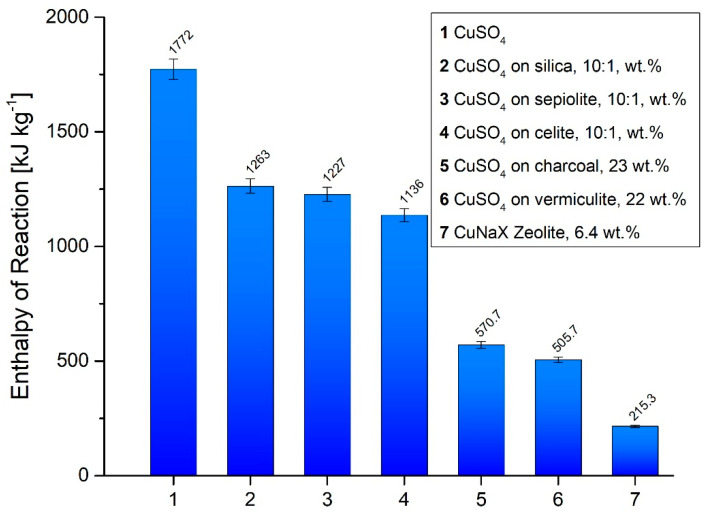
Enthalpy of reaction (energy content) of pure CuSO_4_ (entry 1, [23]) and those corresponding to the samples of different composite materials. Entries 2 to 4 correspond to CuSO_4_ co-precipitated from the suspension of fine-grained matrix material in water. Entries 5 to 6 are the composite carrier materials prepared from impregnation with dissolved CuSO_4_.

The formation of [Cu(NH_3_)_4_]SO_4_ starting from CuSO_4_ provides an enthalpy of reaction of 1.77 MJ kg^−1^ [24]. Upon immobilization on an inert matrix carrier, or by being diluted within the microporous structure of the host material, a notable decrease in the enthalpy of the reaction compared to the pure material was to be expected, as the reactive component per mass is decreased. In the DSC/TGA setup, the enthalpy of reaction was determined for all materials starting from room-temperature, thus being comparable to the conditions used previously for pure CuSO_4_ samples. The results of the corresponding measurements for the various CuSO_4_-composite materials are shown in Figure 2.

**Figure 3:**
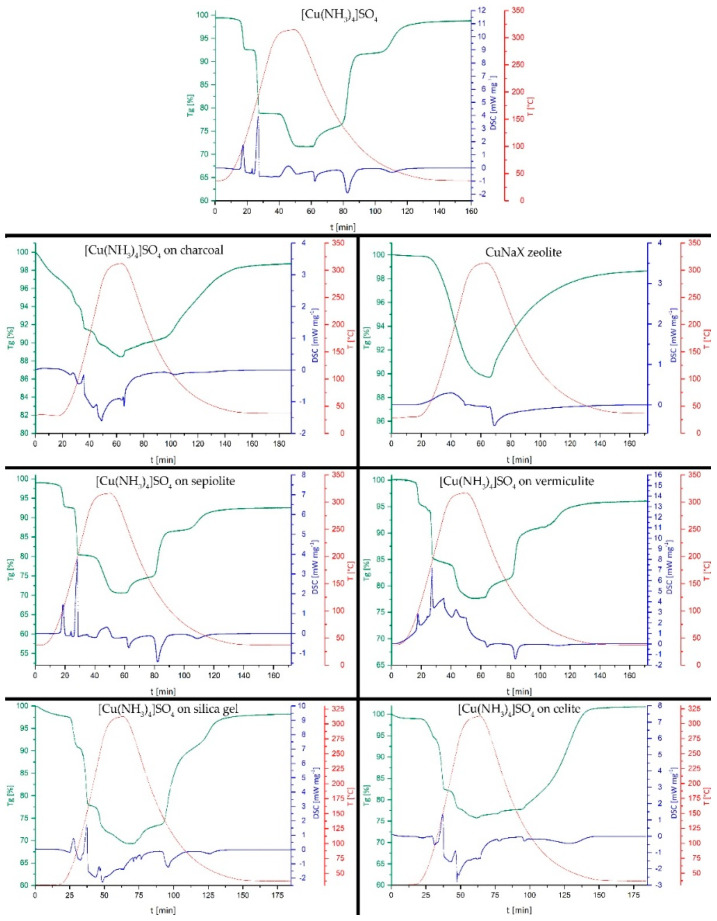
Initial study on reversibility by performing a complete thermal charging–discharging cycle for all various composites. For comparison, the equivalent NH_3_ charging–discharging of pure [Cu(NH_3_)_4_]SO_4_ is given on top. An X-ray powder diffraction (P-XRD) comparison for all composites before and after reaction with NH_3_ are given in the supplementary materials, Figures S2–S6. Comparing the support materials after loading and ammoniation with the P-XRD pattern for pure CuSO_4_ and [Cu(NH_3_)_4_]SO_4_ shows good agreement. Nevertheless, a detailed structural analysis of eventual changes of the support material or interactions with the storage material based on the powder patterns is impossible, due to non-existing comparability with previously reported structures/patterns. This would also exceed the scope of this study.

The entries 2 to 4 correspond to CuSO_4_ as formed by evaporation of suspensions of carrier particles in an aqueous solution of CuSO_4_ at a given weight ratio 1:10. The enthalpy of reaction is affected only marginally by the type of the inert matrix particles, differing between the highest (silica gel, entry 2, 1.26 MJ kg^−1^) and the lowest (celite, entry 4, 1.14 MJ kg^−1^) only by about 10.1%. Compared to the pure material, the dilution with the support causes a drop of around 30% of the initial energy storage capacity. For the entries 5 to 7, the huge disadvantage of these composite materials becomes evident. Due to the notably decreased amount of active thermochemical storage material in the composites (23 wt. % for charcoal, entry 5, to 16 wt. % for zeolite 13X, entry 7), only a small percentage of the initial storage capacity is retained. Compared to the pure material, only between 32.2% (charcoal, entry 5, 0.57 MJ kg^−1^) and 12.2% (zeolite 13X, entry 7, 0.21 MJ kg^−1^) of the initial energy is released during the ammoniation process. It should be mentioned that the investigated support materials are known to absorb ammonia. Accounting for this behavior, reference experiments of previously wetted and afterwards dried support materials were run under NH_3_ atmosphere in the DSC, thus simulating the preparation steps of CuSO_4_ loading. These showed that the NH_3_ uptake was negligible in comparison to the reaction enthalpy liberated during the ammoniation of CuSO_4_. As can be seen from Figure 3, zeolite 13X represents the only exception in the series (Figure 3, line 2 right).

The enthalpy of reaction is not the only relevant aspect when characterizing a potential thermochemical storage material. Both the ammoniation and deammoniation are fully reversible for pure anhydrous CuSO_4_. In order to elucidate whether this behavior is also retained for the various composite materials, an initial assessment was performed by subjecting the composites to a complete thermal charging–discharging cycle under an NH_3_-atmosphere in the DSC/TGA.

All samples of various CuSO_4_-composites were fully ammoniated in-situ before starting the deammoniation-ammoniation cycles shown in Figure 3. The initial mass increase due to the ammonia uptake is not shown in Figure 3. To allow for better comparability, in the first row of Figure 3, the cycling behavior of pure [Cu(NH_3_)_4_]SO_4_ is given, featuring a characteristic stepwise NH_3_ release during thermal decomposition and uptake on subsequent cooling under an NH_3_-atmosphere. The three mass-change steps correspond to one NH_3_ ligand, followed by two NH_3_ ligands and, finally, the last one NH_3_ out of, in total, four ligands, in both directions, as determined by the release and consecutive uptake of NH_3_ on heating and cooling.

For the charcoal-[Cu(NH_3_)_4_]SO_4_ composite, the decomposition occurs in less pronounced steps, as the stepwise removal of the NH_3_-ligands is overlaid by a gradual mass-loss with increasing temperature. For the ammoniation reaction, only one onset of the reaction at 125 °C (see Table 1) is observed. The loaded zeolite (Figure 3, 2nd row right) displays a distinct gradual one-step mass-loss during the deammoniation, starting from 46 °C. The ammoniation reaction starts by a gradual mass-increase at 288 °C. This sagging single-step behavior is indicative of the desorption/adsorption of NH_3_ on the zeolite matrix, instead of a coordination reaction between NH_3_ and previously immobilized CuSO_4_, as seen for the other host materials. This is attributed to the ion-exchange of Cu^2+^ for Na^+^ in the zeolite structure, occurring immediately during the obvious exchange reaction of zeolite 13X in contact with the saturated CuSO_4_ solution. Therefore, the zeolite investigated herein is a partially ion-exchanged CuNaX zeolite with a negligible amount of free reactive CuSO_4_ species on its surface. Such ion exchange reactions, which determine the properties and influence the capabilities of thermochemical energy storage composite materials, are often neglected, but have been described in detail by Gläser et al., 2016 [35].

Both phyllosilicate-based composites formed with sepiolite and vermiculite reveal the characteristic three-step removal and uptake of NH_3_ during thermal cycling. In addition, the onset temperatures for the single steps are also in good agreement with the values measured for the pure material. Both samples show a minor mass-loss over the first cycle, which may be attributed to the residual contents of intercalated water molecules between silicate layers.

The decomposition steps for [Cu(NH_3_)_4_]SO_4_ in silica composites are less pronounced than those found in the pure material, which becomes even more evident when the second and third ammoniation step seem to merge without a stable plateau on cooling. Within the experiment, a slightly higher mass loss than for the pure [Cu(NH_3_)_4_]SO_4_ sample was observed. Since, during cooling in the reaction with NH_3_, the final mass increase does not reach 100%, traces of residual moisture were removed during the first heating cycle. For the composite with celite, the decomposition steps become even more gradual, whereas for the ammoniation, only a single onset of the reaction is observed at 122 °C. As both silica and celite had an inferior performance compared to the fibrous phyollosilicate sepiolite, those two materials were excluded from further investigations at this stage.

The onset temperatures for all samples investigated here and shown in Figure 3 are summarized in Table 2, which also reports the temperature intervals of the onset-temperatures for the corresponding steps during ammoniation and deammoniation, given as the Δ-value. In case only a gradual mass-loss/mass-increase was observed, a single onset-temperature is given.

**Figure 4:**
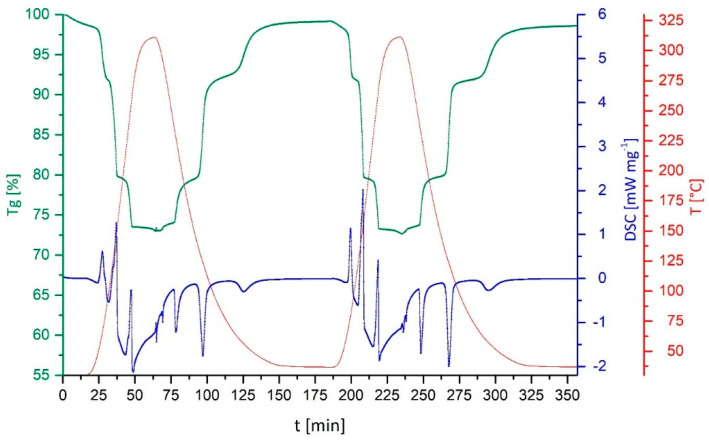
Consecutive charging–discharging cycles number 2 and 3 for [Cu(NH_3_)_4_]SO_4_ in a sepiolite matrix under an NH_3_-atmosphere.

Combining the previously determined reaction enthalpies and the outcome of the charging–discharging cycle, the composite materials with sepiolite and vermiculite are the most promising. Therefore, after the first cycle shown in Figure 3, these two materials were subjected to two further consecutive charging–discharging cycles in the DSC/TGA setup under the NH_3_-atmosphere. It goes without saying that, for a real application, three cycles are by no means representative of a long-term application. For this first study of CuSO_4_ on support materials, however, more than three cycles would have gone beyond its scope. In a next step, long-term cycling performance should be investigated without the time-consuming DSC/TGA setup. In Figure 4, the second and third cycle for [Cu(NH_3_)_4_]SO_4_ on sepiolite are shown.

With respect to the two notable key observations derived from this further cycling of [Cu(NH_3_)_4_]SO_4_ in a sepiolite matrix, one of them addresses the full reversibility. Although limited to only three cycles for the initial verification of the reversibility, the material shows no detectable ageing effects during these first few thermal cycles. However, this finding needs to be considered with caution, as three cycles only provide a limited information value with respect to the long-term behavior. Nonetheless, they are consistent and representative for the short-term behavior. The second major aspect refers to the stepwise removal of the NH_3_-ligands as compared between the second and third cycles. More pronounced steps and clearer intermediate plateaus can be seen with increasing repetition from cycle to cycle. This effect is attributed to the cracking of the layer of the CuSO_4_/[Cu(NH_3_)_4_]SO_4_ during the first cycle, allowing for better accessibility of the material by NH_3_ (see below).

**Figure 5:**
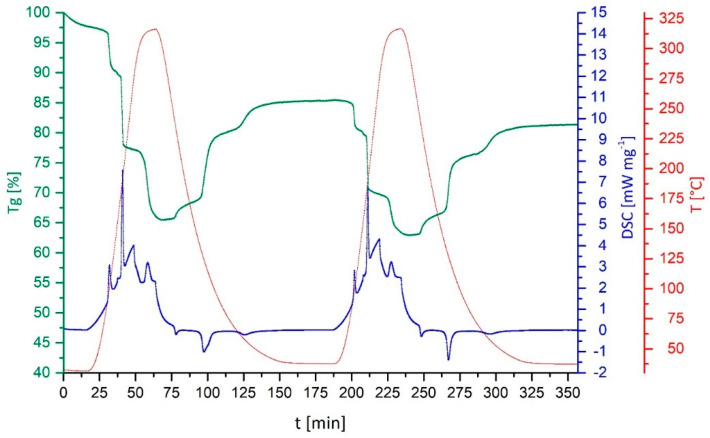
Consecutive charging–discharging cycles 2 and 3 for [Cu(NH_3_)_4_]SO_4_ on vermiculite under an NH_3_-atmosphere.

In Figure 5, the second and third cycles for [Cu(NH_3_)_4_]SO_4_ on vermiculite are shown.

During the further cycling of the [Cu(NH_3_)_4_]SO_4_ + vermiculite composite, the overall mass-loss observed already within the first cycle (see Figure 3) continues, reaching 18.6% after the third cycle. This further loss cannot be attributed simply to the evaporation of moisture. The characteristic steps in the TG curve remain unchanged and, thus, this mass-decrease must be attributed to the vermiculite matrix, most likely due to structurally intercalated volatile components. Further cycles could show whether a slow decomposition of the matrix would also affect the charging–discharging behavior of the thermochemical storage material on a longer perspective, or if the change in the composite material seizes during further thermal treatment. 

### 3.2. Thermal Conductivity

**Figure 6:**
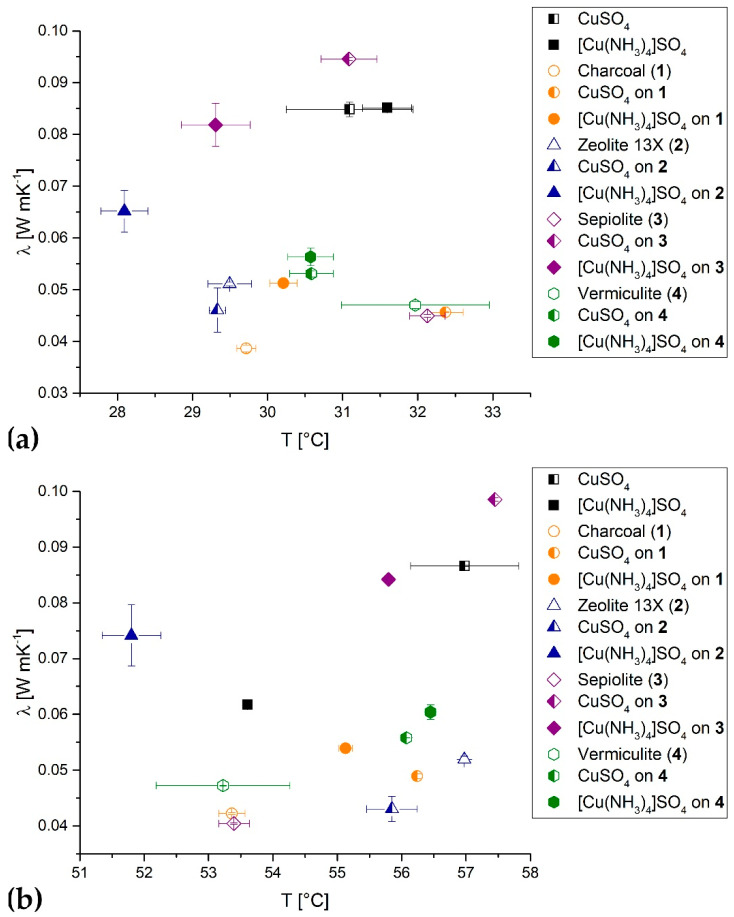
Comparison of the thermal conductivity for all CuSO_4_ and [Cu(NH_3_)_4_]SO_4_ composites: (**a**) thermal conductivity at 30 °C, (**b**) thermal conductivity at 55 °C. The results for the pure salts are represented in black, for charcoal (in the legend stated as (**1**) in orange, for zeolite 13X (**2**) in blue, for sepiolite (**3**) in violet and for vermiculite (**4**) in green.

In order to derive whether the CuSO_4_-composite materials feature an improved thermal conductivity in comparison to the pure bulk sulfate salt material, comparative transient hot bridge measurements for all materials were performed. For all available composite materials, both forms with NH_3_-free CuSO_4_ and ammoniated [Cu(NH_3_)_4_)SO_4_ were measured, in order to compare the difference in the effective thermal conductivity between the charged and discharged state of the bulk. Although a determination of the changing thermal conductivity over temperature following a complete charging–discharging cycle would be of interest, experiments were limited to two measurement series at 30 °C and 55 °C due to the corrosivity of NH_3_ (as originates from beginning deammoniation over 70 °C). In Figure 6, the effective thermal conductivity of the bulk of all materials is compared (Figure 6a at 30 °C, Figure 6b at 55 °C).

Pure CuSO_4_ and [Cu(NH_3_)_4_)SO_4_ exhibit a highly comparable effective thermal conductivity of 0.085 W mK^−1^ at 30 °C (Figure 6a, black squares). The lowest effective thermal conductivities were measured for the original untreated carrier materials, ranging from 0.039 W mK^−1^ (charcoal) to 0.051 W mK^−1^ (zeolite 13X). Once the host carrier materials are loaded with CuSO_4_ or [Cu(NH_3_)_4_)SO_4_, their effective thermal conductivity increases notably. The only exception to this behavior is found for CuSO_4_ in combination with the zeolite matrix, seemingly decreasing the effective thermal conductivity compared to the pure zeolite. However, this apparent decrease is afflicted with a certain experimental error, relativizing this observation. Aiming for an improved thermal conductivity of the composite materials, compared to the pure storage material only CuSO_4_ and [Cu(NH_3_)_4_]SO_4_ on sepiolite (Figure 6a entry 3, violet squares) are in the same range. Nevertheless, the largest effective thermal conductivity measured at 30 °C was obtained for the sepiolite-CuSO_4_ composite, revealing a value of 0.095 W mK^−1^.

For the measurements at 55 °C, a larger dispersion can be observed for all materials, and a notable difference can also be observed between CuSO_4_ and [Cu(NH_3_)_4_]SO_4_. The effective thermal conductivity of CuSO_4_ (0.087 W mK^−1^) is 29% higher than for the tetrammine-complex (0.062 W mK^−1^). At 55 °C, the effective thermal conductivity of the support materials did not change significantly compared to 30 °C. Again, CuSO_4_ and [Cu(NH_3_)_4_]SO_4_ on sepiolite (Figure 6b entry 3, violet squares) showed the largest thermal conductivities out of all composite materials, representing a remarkable improvement in relation to the pure salts.

Thermal conductivity measurements do not show an improvement in properties through the deposition of CuSO_4_ on any host carrier materials. Any thermal conductivity enhancement observed for the composite materials is in no relation to the loss of storage density due to the dilution of the reactive copper sulfate species within the host material. However, two issues associated with the enhancement of the thermal conductivity are worth mentioning. First, there are materials even better suited for preparing composite materials with enhanced thermal conductivity which have not been considered for this study. For example, expanded natural graphite features significantly higher thermal conductivities even as a pure material [36]. Second, the accuracy of the transient hot bridge method is strongly dependent on the contact between the material and the sensor. For powdered materials such as pure salts or the sepiolite samples, the contact is much better than, e.g., for the zeolite samples, with a much lower bulk density and contact area to the sensor. This affects the outcome significantly and should therefore be kept in mind. Nevertheless, the results obtained by this method are a reliable and appropriate measure of the effective thermal conductivity of the bulk materials.

### 3.3. Scanning Electron Microscopy (SEM)

**Figure 7:**
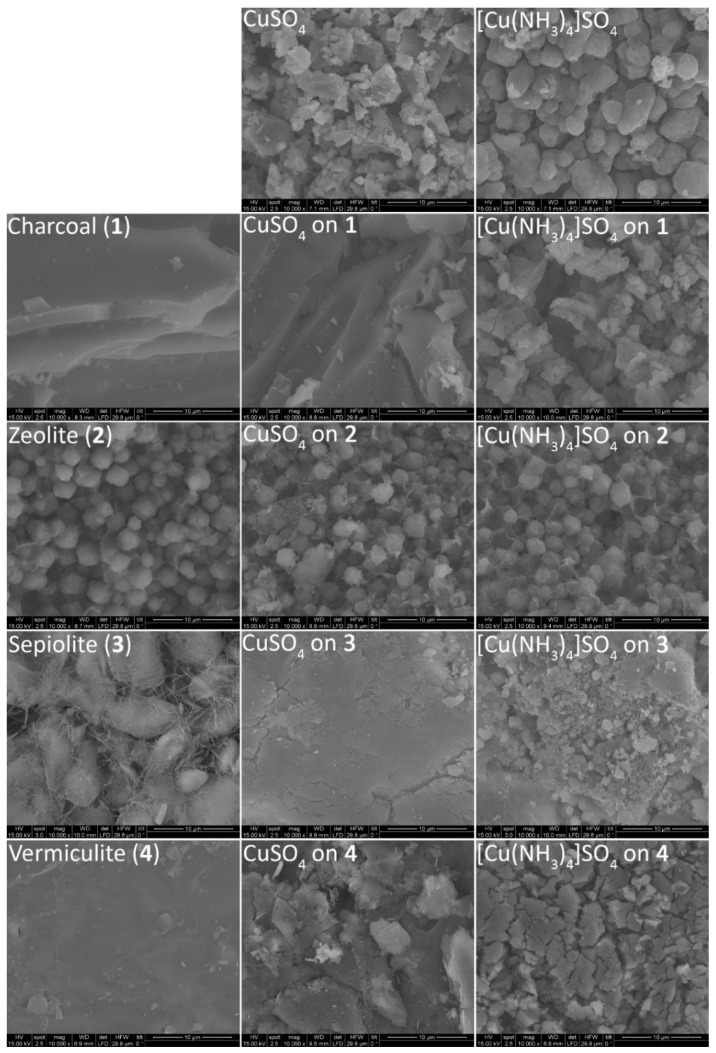
SEM-images of the pristine support materials (left column), CuSO_4_ loaded to the support materials (middle column) and the loaded support materials after reaction with NH_3_ (right column). On top SEM-images of CuSO_4_ and [Cu(NH_3_)_4_]SO_4_ are given for comparison. All images were taken ad a 10,000-fold magnification, the white bar corresponds to a length of 10 µm.

In order to get a better a better understanding of how the studied composite materials change regarding their particle morphology during the respective reactions, SEM images of the pristine carrier materials and images of the composites are compared. As a reference in Figure 7, the particle morphology both for pure CuSO_4_ and [Cu(NH_3_)_4_]SO_4_ is shown.

The SEM image of CuSO_4_ shows the material in form of agglomerated platelets with varying particle size, the largest having an approximate diameter of 4 µm. After reaction with NH_3_, the volumetric expansion is also evident from the morphology of the [Cu(NH_3_)_4_]SO_4_ particles. The previous variety in the particle size distribution, idiomorphically shaped with clear crystal faces and edges, has changed towards more uniform xenomorphic particles, at an equivalent size around 4–5 µm in diameter.

For the untreated charcoal used as carrier material, large stacked layers dominate the morphology. Treatment with CuSO_4_ results in a morphology highly comparable to the pristine sample, showing dispersed CuSO_4_ crystallites and particles deposited on the surface of the charcoal. Reaction with NH_3_ changes the morphology completely, as now the whole surface of the charcoal is covered with small [Cu(NH_3_)_4_]SO_4_ particles. The shape of these particles is very similar to that of the pure tetraammine-complex.

The microstructure of the pristine zeolite 13X is dominated by spherical agglomerates of a rather narrow size distribution. On treatment with CuSO_4_ solution a few smaller particles on the surface of the zeolite grains become visible, apparently not affected after the reaction with NH_3_. The particle morphology of the zeolite 13X samples is largely unaffected by the treatment with CuSO_4_ and subsequent reaction with NH_3_. Nevertheless, the SEM images provide no information about the amount of Cu^2+^ and sulfate being located inside the zeolite microstructure as a consequence of ion exchange within the aqueous solution.

Pure sepiolite shows bulk particles of a soft-spherical texture with diameters around 10 µm, which are overlaid by a fine fibrous structure in between the larger particles. On impregnation with CuSO_4_ this structure is completely lost, changing towards flat agglomerates of fine particles. Reaction with NH_3_ results in an increase in the particle size, resulting in agglomerates of sheds.

Vermiculite shows a smooth surface texture in the SEM, which is fragmented on the treatment with the CuSO_4_ solution. This might be due to the swelling properties and intercalation between silicate layer units. On the surface, large agglomerates of fine CuSO_4_-particles appear, cracking the smooth vermiculite underneath. After reaction with NH_3_, the complete surface is covered by cracked agglomerates, which appear to originate from the excess volume of the formation of complexes in [Cu(NH_3_)_4_]SO_4_.

**Figure 8:**
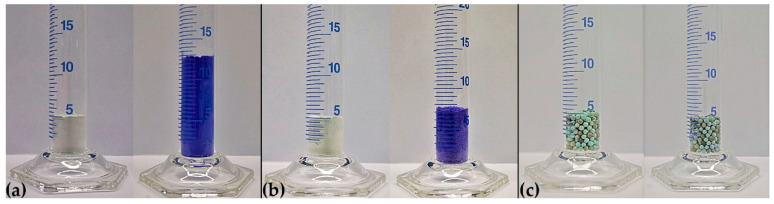
Volumetric expansion of CuSO_4_ during reaction with NH_3_, resulting [Cu(NH_3_)_4_]SO_4_ in the case of (**a**) pure CuSO_4_, (**b**) CuSO_4_ on sepiolite 10:1 wt. % and (**c**) CuNaX zeolite. For the zeolite sample the color change during reaction with NH_3_ towards the darker color of [Cu(NH_3_)_4_]SO_4_ is hardly visible.

Having compared all samples, it becomes obvious that, apart from the zeolite on all host carrier materials, the reaction with NH_3_ causes a notable change in morphology based on the above-mentioned volume work. Although still visible on a morphological level, in comparison to the 2.6-fold expansion of the pure CuSO_4_ during ammoniation, the deposition on an inert support material could notably decrease the expansion on a bulk scale. In Figure 8, the reaction of pure CuSO_4_ (Figure 8a), CuSO_4_ on sepiolite (Figure 8b) and CuNaX-zeolite (Figure 8c) is shown.

From the 2.6-fold expansion, the 10:1 wt. % dilution with sepiolite already reduced the expansion to the 1.3-fold volume; whereas in the case of zeolite, no volume work during ammoniation occurred. This comparison underlines the fact that the choice of the inert matrix material can actively contribute to solving the issue of volume expansion in the system CuSO_4_/[Cu(NH_3_)_4_]SO_4_.

## 4. Conclusions

The thermochemical energy storage system CuSO_4_/[Cu(NH_3_)_4_]SO_4_ combines an energy storage density of 1.77 MJ kg^−1^ with the complete reversibility of the reaction, thus being a promising material for technological applications. The volume work between the charged and discharged state, amounting to a 2.6-fold expansion or shrinkage, could affect applicability. Therefore, a study on various composite materials, formed by combining CuSO_4_ on charcoal, vermiculite, zeolite 13X, sepiolite, silica (SiO_2_) and celite, focusing on their basic thermochemical parameters (energy storage density and reversibility) as well as on their bulk expansion and thermal conductivity, was performed. 

The key results of this study are:

(1) The formation of composites of CuSO_4_ with silica, sepiolite and celite (10:1 wt. %) results in energy storage densities between 1.14–1.26 MJ kg^−1^, around 30 % lower than for the pure salt. Adsorption and intercalation of CuSO_4_ on charcoal, in vermiculite and zeolite 13X substrate materials decreased the energy storage to 12.2 % (zeolite 13X) and 32.2 % (charcoal) of the initial value. For the zeolite, an ion exchange of Na^+^ to Cu^2+^ occurs, resulting a mixed CuNaX-zeolite, only capable of NH_3_^−^ adsorption and desorption on the Cu-sites.
(2) Only CuSO_4_ on sepiolite and on vermiculite retained the characteristic stepwise uptake and removal of the NH_3_-ligands. In terms of cycle stability, the composite with sepiolite provides full reversibility of the reaction, for the composite with vermiculite during the three investigated cycles, a certain mass-loss was observed as attributed to partial decomposition of the vermiculite substrate.
(3) The effective thermal conductivities of the materials, determined at 30 °C and 55 °C, were not really improved compared to the pure CuSO_4_/[Cu(NH_3_)_4_]SO_4_. Only CuSO_4_ on sepiolite, especially at 55 °C, provides a higher effective thermal conductivity in the bulk.
(4) Through the immobilization of the thermochemical energy storage material on the support/composite formation, a small reduction in energy content is traded for a drastically reduced volume change. The bulk volume expansion during reaction with NH_3_ is reduced for the sepiolite composite to a 1.3-fold expansion, as the composite material compensates for most of the molecular expansion during the reaction with NH_3_. Charcoal, vermiculite and CuNaX zeolite composites show no volume work during the reaction with NH_3_, the CuSO_4_ being incorporated in the matrix support.
(5) Within further steps, a long-term cycling study of the sepiolite composite is suggested. Furthermore, other composite materials, presumably with better thermal conductivities (e.g., expanded natural graphite), should be investigated.

## Figures and Tables

**Table 1:** Specific surface area, pore volume and average pore size for the selected host/support materials.

	Specific Surface Area [m^2^ g^−1^]	Pore Volume [cm^3^ g^−1^]	Average Pore Size [Å]
**Celite**	0.1570 ± 0.02	n.d.	n.d.
**Charcoal**	1694 ± 34	1.47 ± 0.03	34.775 ± 0.003
**Sepiolite**	709 ± 13	1.34 ± 0.09	76.046 ± 0.001
**Silica**	670 ± 2	0.95 ± 0.03	56.71 ± 0.012
**Vermiculite**	0.55 ± 0.19	n.d.	n.d.
**Zeolite 13X**	525 ± 16	n.d.	n.d

**Table 2:** Deammoniation/ammoniation temperatures for [Cu(NH_3_)_4_]SO_4_ on support materials corresponding to Figure 3. All temperatures were obtained by evaluation with the software-package and are attributed with an uncertainty of ±2 °C.

	**bulk [Cu(NH_3_)_4_]SO_4_**					
	**↑ [°C]**	**↓ [°C]**	**Δ [°C]**			
1st step	79	66	13			
2nd step	168	138	30			
3rd step	307	248	59			
	**[Cu(NH_3_)_4_]SO_4_ + charcoal**			**CuNaX zeolite**		
	**↑ [°C]**	**↓ [°C]**	**Δ [°C]**	**↑ [°C]**	**↓ [°C]**	**Δ [°C]**
1st step	65	---	---	46	---	---
2nd step	119	---	---	---	---	---
3rd step	212	125	87	---	288	---
	**[Cu(NH_3_)_4_]SO_4_ + sepiolite**			**[Cu(NH_3_)_4_]SO_4_ + vermiculite**		
	**↑ [°C]**	**↓ [°C]**	**Δ [°C]**	**↑ [°C]**	**↓ [°C]**	**Δ [°C]**
1st step	77	74	3	85	96	−11
2nd step	170	138	32	171	129	42
3rd step	302	265	37	305	236	69
	**[Cu(NH_3_)_4_]SO_4_ + silica gel**			**[Cu(NH_3_)_4_]SO_4_ + celite**		
	**↑ [°C]**	**↓ [°C]**	**Δ [°C]**	**↑ [°C]**	**↓ [°C]**	**Δ [°C]**
1st step	104	85	19	106	---	---
2nd step	210	134	76	190	---	---
3rd step	294	267	27	284	122	162

## Supplementary Materials

The following are available online at https://www.mdpi.com/2079-4991/10/12/2485/s1, Figure S1: Images of CuSO_4_ on materials before and after reaction with NH_3_, Figure S2: Comparison of the P-XRD pattern for CuSO_4_ and [Cu(NH_3_)_4_]SO_4_, Figure S3: Comparison of the P-XRD pattern for charcoal, CuSO_4_ on charcoal and [Cu(NH_3_)_4_]SO_4_ on charcoal, Figure S4: Comparison of the P-XRD pattern for sepiolite, CuSO_4_ on sepiolite and [Cu(NH_3_)_4_]SO_4_ on sepiolite, Figure S5: Comparison of the P-XRD pattern for vermiculite, CuSO_4_ on vermiculite and [Cu(NH_3_)_4_]SO_4_ on vermiculite, Figure S6: Comparison of the P-XRD pattern for zeolite 13X, CuNa zeolite 13X and CuNa-zeolite 13X after reaction with NH_3_.
