# Supplementary material for: CuSO4/[Cu(NH3)4]SO4-Composite Thermochemical Energy Storage Materials
Source: Nanomaterials (Basel). 2020 Dec 11;10(12):2485. doi: 10.3390/nano10122485 (PMC7763518; doi:10.3390/nano10122485)
Supplement: Supplementary file 1 [file nanomaterials-10-02485-s001.pdf]

nanomaterials

Supporting Information

$CuSO_4$/[$Cu(NH_3)_4$]SO_4-Composite Thermochemical Energy Storage Materials

Danny Müller [1,*], Christian Knoll [1,2], Georg Gravogl [1,3], Daniel Lager [4], Jan M. Welch [5], Elisabeth Eitenberger [6], Gernot Friedbacher [6], Andreas Werner [7], Werner Artner [8], Michael Harasek [2], Ronald Miletich [3] and Peter Weinberger [1,*]

1 Institute of Applied Synthetic Chemistry, TU Wien, Getreidemarkt 9, 1060 Vienna, Austria; christian.knoll@gmx.at (C.K.); georg.gravogl@tuwien.ac.at (G.G.)
2 Institute of Chemical, Environmental & Biological Engineering, TU Wien, Getreidemarkt 9, 1060 Vienna, Austria; michael.harasek@tuwien.ac.at (M.H.)
3 Institut für Mineralogie und Kristallographie, University of Vienna, Althanstraße 14, 1090 Vienna, Austria; ronald.miletich-pawliczek@univie.ac.at (R.M.)
4 Austrian Institute of Technology GmbH, Giefinggasse 2, 1210 Vienna, Austria; daniel.lager@ait.ac.at (D.L.)
5 Center for Labelling and Isotope Production, TRIGA Center Atominstitut, TU Wien, Stadionallee 2, 1020 Vienna, Austria; jan.welch@tuwien.ac.at (J.W.)
6 Institute of Chemical Technologies and Analytics, TU Wien, Getreidemarkt 9, 1060 Vienna, Austria; elisabeth.eitenberger@tuwien.ac.at (E.E.); gernot.friedbacher@tuwien.ac.at (G.F.)
7 Institute for Energy Systems and Thermodynamics, TU Wien, Getreidemarkt 9, 1060 Vienna, Austria; andreas.werner@tuwien.ac.at (A.W.)
8 X-Ray Center, TU Wien, Getreidemarkt 9, 1060 Vienna, Austria; email: werner.artner@tuwien.ac.at (W.A.)
* Correspondence: danny.mueller@tuwien.ac.at (D.M.); peter.e163.weinberger@tuwien.ac.at (P.W.); Tel.: +43-1-58801-163740 (D.M.); +43-1-58801-163617 (P.W.)

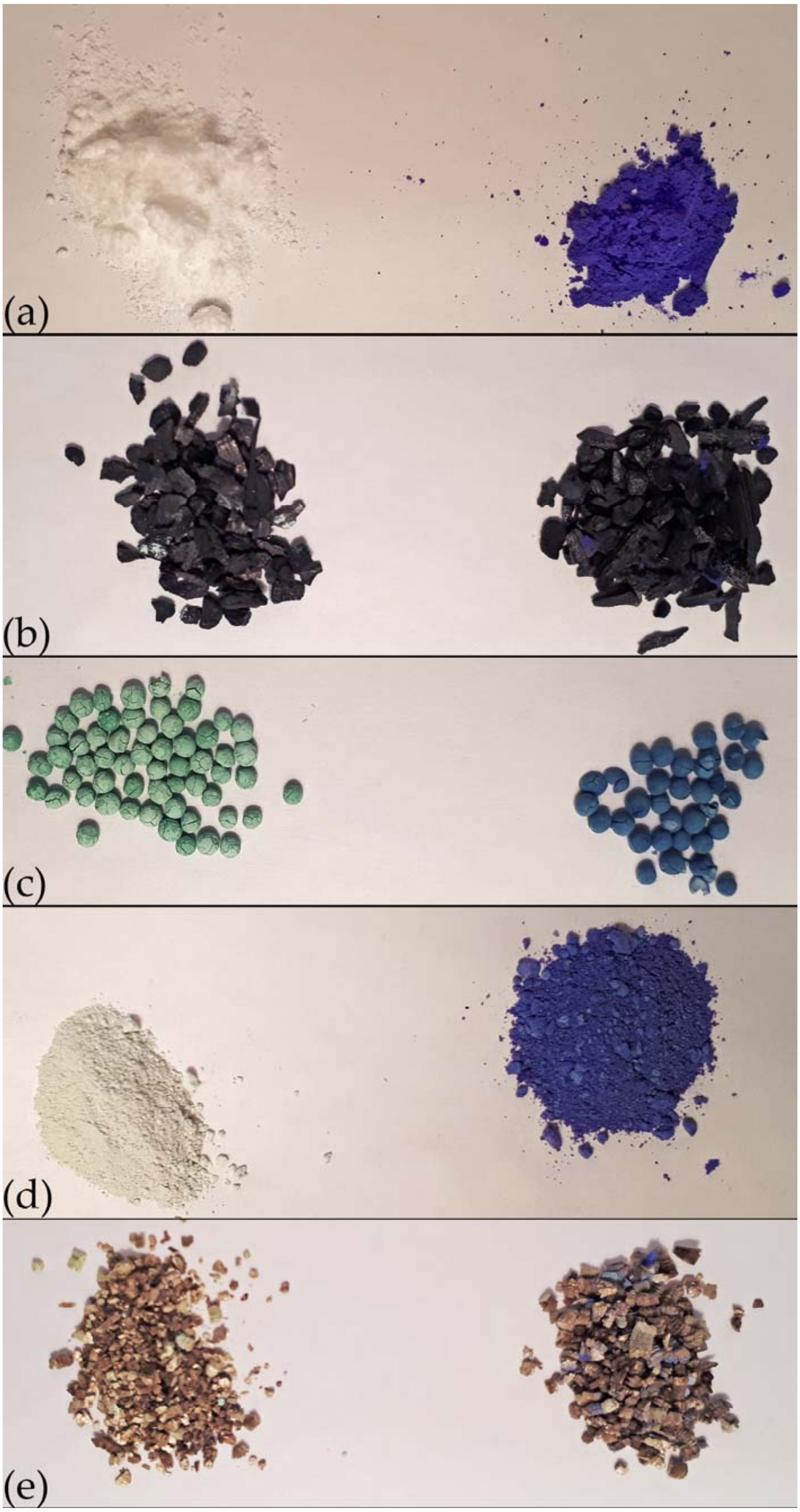

Figure S1. (**a**) CuSO4 (left) and [Cu(NH3)4]SO4 (right); (**b**) CuSO4 on charcoal (left) and [Cu(NH3)4]SO4 on charcoal (right); (**c**) CuNa-zeolite 13X (left) and CuNa-zeolite 13 after reaction with NH3 (right); (**d**) CuSO4 on sepiolite (left) and [Cu(NH3)4]SO4 on sepioite (right); (**e**) CuSO4 on vermiculite (left) and [Cu(NH3)4]SO4 on vermiculite (right);.

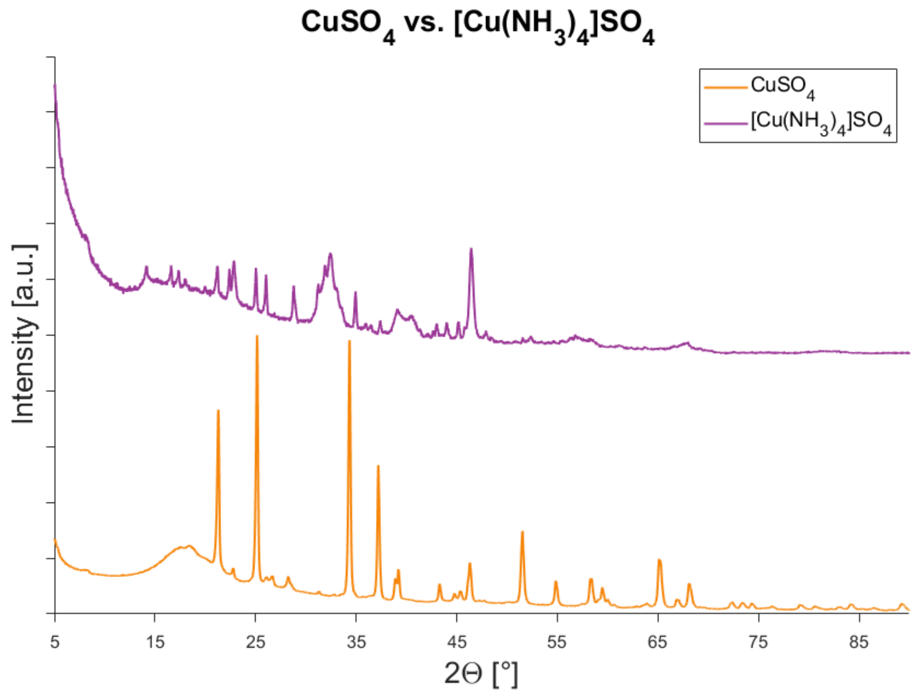

Figure S2. Comparison of the P-XRD pattern for $CuSO_4$ and $[Cu(NH_3)_4]SO_4$.

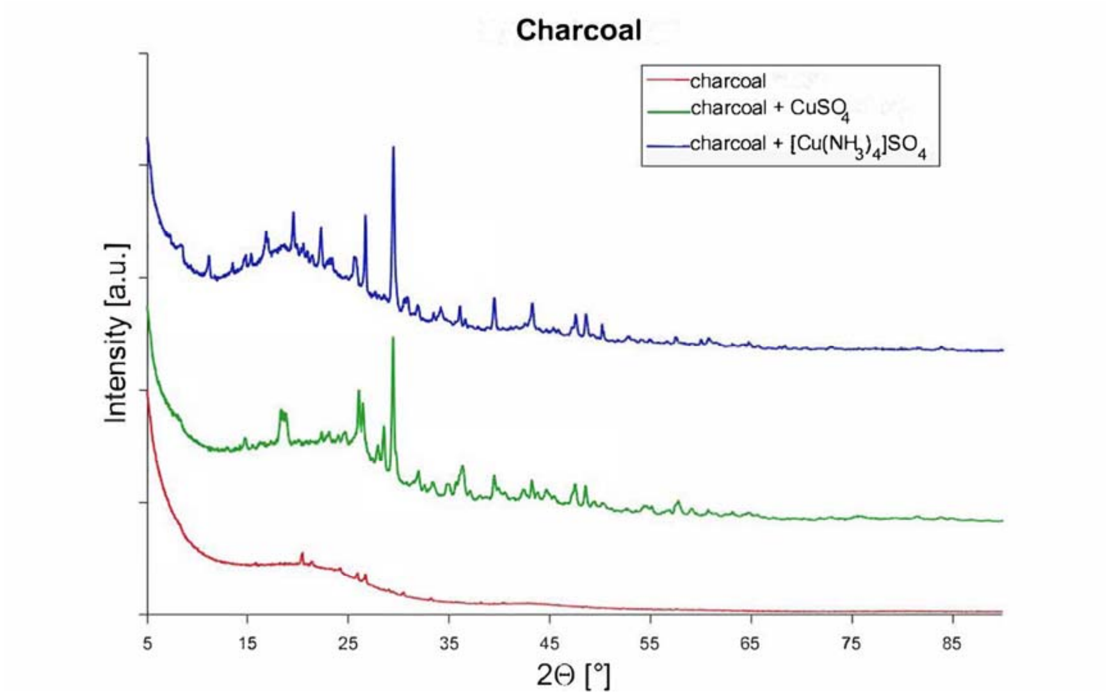

Figure S3. Comparison of the P-XRD pattern for charcoal, $CuSO_4$ on charcoal and $[Cu(NH_3)_4]SO_4$ on charcoal.

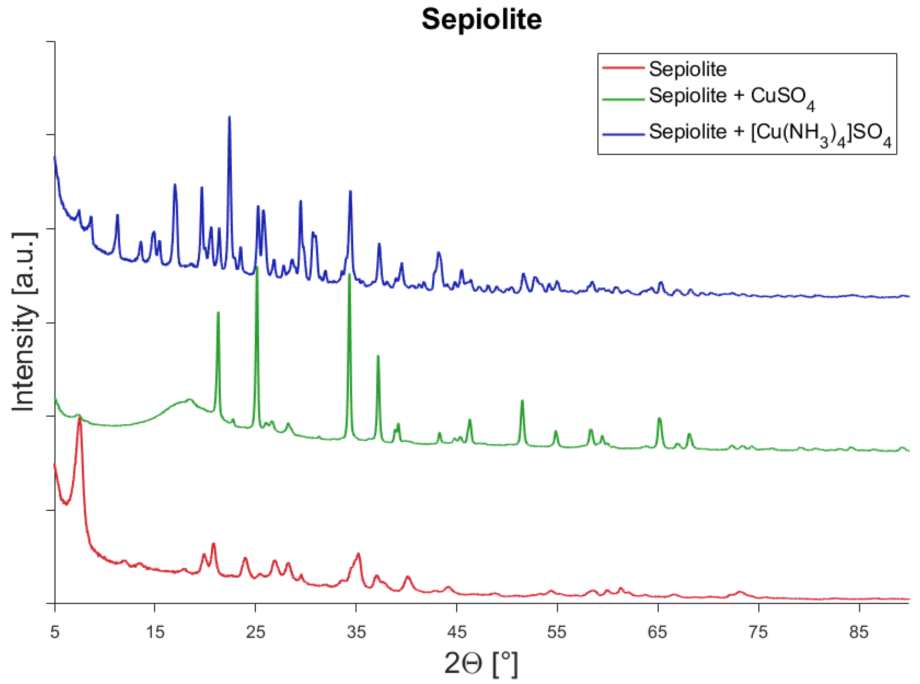

Figure S4. Comparison of the P-XRD pattern for sepiolite, $CuSO_4$ on sepiolite and $[Cu(NH_3)_4]SO_4$ on sepiolite.

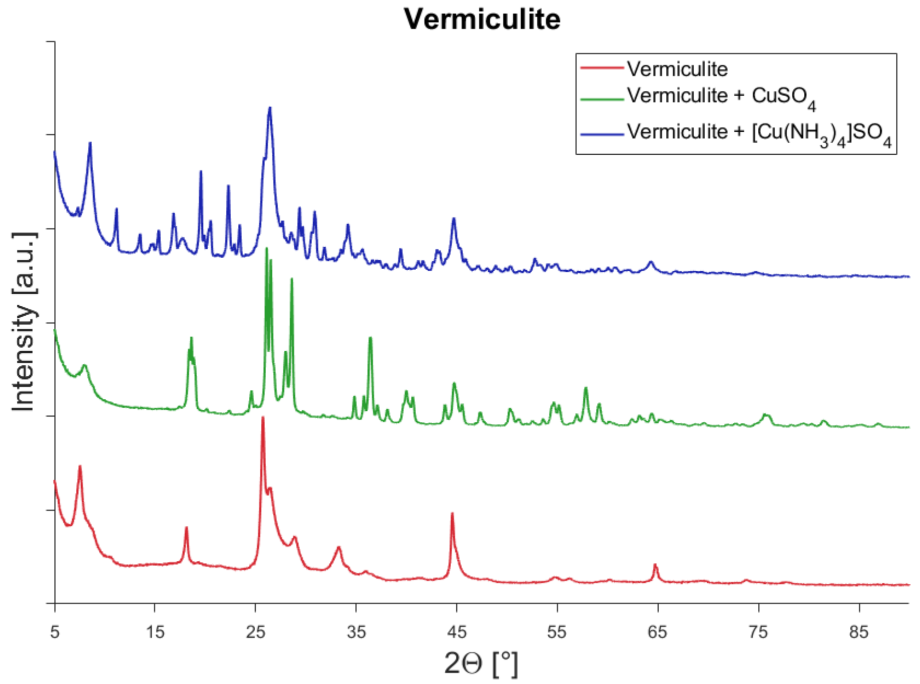

Figure S5. Comparison of the P-XRD pattern for vermiculite, $CuSO_4$ on vermiculite and $[Cu(NH_3)_4]SO_4$ on vermiculite.

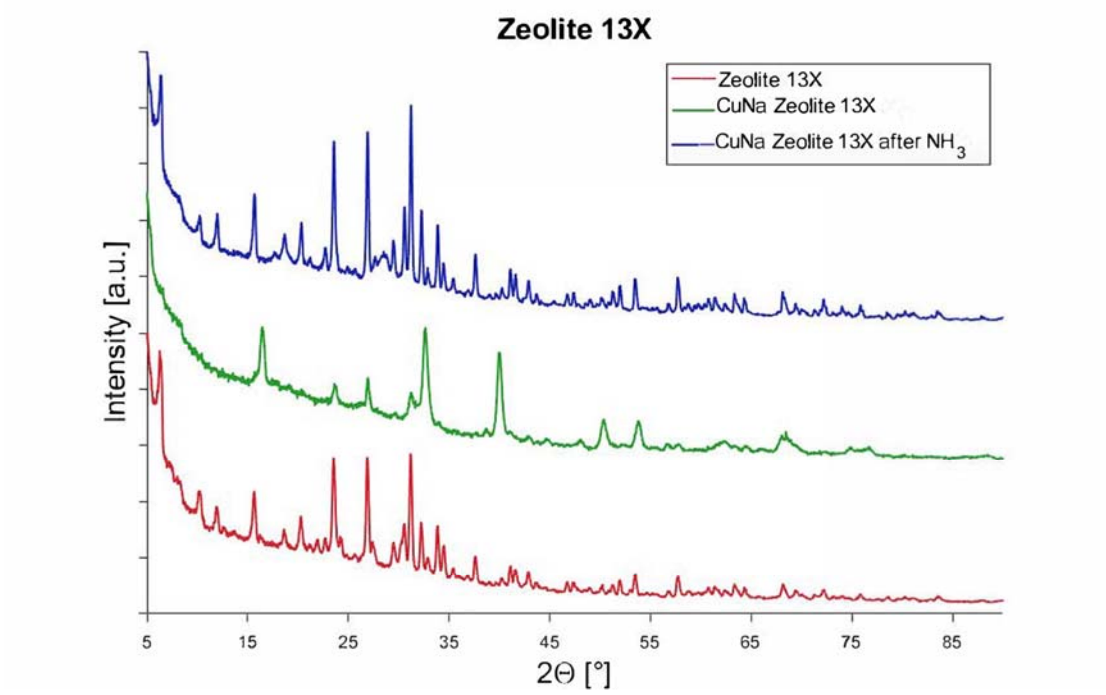

Figure S6. Comparison of the P-XRD pattern for zeolite 13X, CuNa zeolite 13X and CuNa-zeolite 13X after reaction with NH_3.
